# Whole‐Brain Neural Connectivity to Cholinergic Neurons in the Lower Thoracic Intermediolateral Column

**DOI:** 10.1002/cns.70902

**Published:** 2026-04-27

**Authors:** Yuan‐jun Yang, Kai‐ying Zhang, Bin‐bin Li, Xia‐wan Liu, Jing‐rong Li, Yi‐nuo Liu, Yu‐han Liu, Ji Li, Xiang‐shan Yuan, Ming Zhong

**Affiliations:** ^1^ Department of Critical Care Medicine, Zhongshan Hospital Fudan University Shanghai China; ^2^ Department of Anatomy and Histoembryology, School of Basic Medical Sciences Fudan University Shanghai China; ^3^ Department of Pancreatic Surgery, Huashan Hospital Fudan University Shanghai China; ^4^ Shanghai Key Laboratory of Lung Inflammation and Injury Shanghai China; ^5^ Shanghai Institute of Infectious Disease and Biosecurity, School of Public Health Fudan University Shanghai China

**Keywords:** cholinergic neurons, rabies virus, spinal cord, sympathetic nervous system

## Abstract

**Objective:**

The intermediolateral column (IML) serves as a crucial hub for sympathetic information processing between the brain and peripheral organs, with its defining hallmark being the presence of cholinergic neurons expressing choline acetyltransferase (ChAT). Specifically, the IML of the lower thoracic cord plays a pivotal role in regulating abdominal metabolic and digestive viscera. However, little is known about the whole‐brain neural connectivity targeting these lower thoracic IML cholinergic neurons, necessitating further investigation.

**Methods:**

Specific retrograde tracing virus was injected into the lower thoracic IML of ChAT‐Cre transgenic mice expressing Cre in the cholinergic neurons. After the virus has fully expressed, brain and spinal cord sections were prepared for whole‐brain fluorescence imaging and quantitative analysis.

**Results:**

We found that virally labeled neurons were detected in 40 brain regions of ChAT‐Cre mice, encompassing the telencephalon, diencephalon, and brainstem. Afferents were predominantly concentrated in 25 brainstem regions, with the pons providing the highest total number of afferents and the medulla offering the highest afferent density across both hemispheres. Although a small subset of regions exhibited strictly unilateral inputs or hemispheric preference, the overall projection pattern remained bilateral. Furthermore, our results revealed extensive projections to the IML from regions classically implicated in sympathetic outflow regulation and homeostatic control, including the paraventricular hypothalamic nucleus (PVN), lateral hypothalamic area (LH), and rostroventrolateral reticular nucleus (RVLM). In addition, inputs were also observed from motor‐related regions, such as primary and secondary motor cortices (M1, M2), red nucleus (RN), and gigantocellular reticular nucleus (Gi), suggesting a potential anatomical basis for the central coupling of somatic motor and sympathetic functions.

**Conclusion:**

Our study provides a comprehensive whole‐brain anatomical map of inputs to lower thoracic IML cholinergic neurons. This extensive supraspinal network, integrating classical sympathetic and homeostatic centers with motor‐related regions, suggests a potential anatomical basis for the central coordination of somatic motor and autonomic functions.

## Graphical abstract

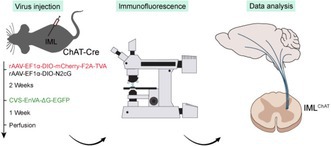

This study establishes a whole‐brain monosynaptic input map to cholinergic neurons in the lower thoracic intermediolateral column (IML) using a cell‐type‐specific retrograde rabies tracing system in ChAT‐Cre mice. Our results reveal a widespread supraspinal network originating from five major brain divisions, including the telencephalon, diencephalon, midbrain, pons, and medulla.

## 1 Introduction

The regulation of peripheral organs and functions by the central nervous system has become a frontier in neuroscience research. Recent studies on interactions between the brain and visceral organs have underscored the critical role of central neural pathways in maintaining systemic homeostasis and behaviors, such as thermoregulation, metabolism, and motor coordination [1, 2, 3]. In this context, the sympathetic nervous system plays an indispensable role in mediating these central regulations over peripheral organs [4]. Therefore, unveiling the precise neural pathways governing sympathetic outflow is essential for a comprehensive understanding of central autonomic control.

The execution of sympathetic functions relies on the transmission of signals through the intermediolateral column (IML), which serves as the final central integration center for sympathetic outflow [5]. Anatomically situated in the thoracolumbar spinal cord segments, the IML bulges laterally to form the lateral horn of the gray matter [6]. It contains the majority of preganglionic sympathetic neurons, which are choline acetyltransferase (ChAT)‐positive neurons. These cholinergic neurons project to peripheral ganglia to innervate organs in a segmental pattern, with rostral segments regulating cardiovascular and respiratory functions, whereas those in the caudal segments target abdominal and pelvic viscera [7, 8, 9]. Within this caudal region, the IML in the 9th to 11th thoracic segments (T9–T11) is particularly critical in regulating abdominal metabolic and digestive functions, including gastric motility, adrenal secretion, and the activity of secretory organs such as the pancreas and biliary system [4, 10, 11]. Accordingly, in the present study, we define the T9–T11 segments as the lower thoracic IML. Given their established role in abdominal sympathetic outflow, this region is critically involved in maintaining metabolic homeostasis and regulating visceromotor activity. Previous research has established that several specific brain regions project to the lower thoracic IML, such as the paraventricular hypothalamic nucleus (PVN), lateral hypothalamic area (LH), and rostroventrolateral reticular nucleus (RVLM) [12, 13, 14]. However, these studies using retrograde tracers such as fluorescent tracers and pseudorabies virus (PRV) had difficulty in ruling out interneuron interference [15] and distinguishing monosynaptic from polysynaptic inputs. Thus, the detailed, whole‐brain afferent landscape specifically targeting lower thoracic IML cholinergic neurons remained to be fully elucidated.

Conventional retrograde transsynaptic viral strategies based on the SAD‐B19 rabies strain are hindered by limitations including low transfer efficiency and high neurotoxicity [16, 17]. Conversely, the novel CVS‐N2c strain exhibits accelerated transport and elevated neuroinvasiveness, allowing for the labeling of long‐range projections [18]. Leveraging this technical advantage, the present study employed the CVS‐N2c strategy in conjunction with ChAT‐Cre mice to systematically map the detailed whole‐brain afferent inputs to lower thoracic IML cholinergic neurons. This work aims to establish a comprehensive neuroanatomical framework, providing a foundation for future functional and interventional studies elucidating the central regulation of peripheral functions.

## 2 Methods

### 2.1 Animals

Five adult ChAT‐Cre mice (male and female, RRID: IMSR_JAX: 018957, C57BL/6J background strain, 2–4 months, 25–27 g) and three wild‐type littermate mice were used in experiments. Mice were housed at 22°C ± 1°C, 55% ± 5% humidity, and under a 12‐h light/dark cycle (lights on at 7 a.m.) with food and water ad libitum.

### 2.2 Viruses and Surgeries

All viral vectors were packaged from BrainCase Biotech (Shenzhen, China) and stored at −80°C for use. The retrograde viruses included CVS‐EnVA‐ΔG‐EGFP (titer 2.0 × 10^8^ genomic copies/mL), rAAV‐EF1α‐DIO‐mCherry‐F2A‐TVA (AAV 9 serotype; titer 2.08 × 10^12^ genomic copies/mL) and rAAV‐EF1α‐DIO‐N2cG (AAV 9 serotype; titer 5.01 × 10^12^ genomic copies/mL).

Mice were anesthetized with 4% isoflurane using a vaporizer, and fur was removed from the back with a shaver. Virus injection was performed according to the methodology established by Haenraets et al. [19]. Via continuous delivery of 1.5% isoflurane with a mask, mice were placed in a stereotaxic holder. The head was tilted forward and the tail was gently elevated and held using a spinal vertebrae clamp (RWD Life Science, Shenzhen, China). To precisely target the T9‐T11 spinal cord segments, we utilized the T13 vertebra as an anatomical reference point, identified by locating its attachment to the most caudal rib pair [20, 21]. Based on the anatomical correspondence between vertebral and spinal levels described by Harrison [22], the T7‐T9 vertebrae were identified as overlying the target spinal segments by counting rostrally from the T13 landmark. A midline incision of approximately 2 cm was made and excess tissue was removed with blunt dissection using forceps under a stereomicroscope, ensuring full exposure of the vertebral column and intervertebral spaces. No laminectomy was performed to minimize trauma, and sterile saline was applied to ensure the exposed tissue remains moist throughout the procedure. Two helper viruses, rAAV‐EF1α‐DIO‐mCherry‐F2A‐TVA and rAAV‐EF1α‐DIO‐N2cG, were mixed at a ratio of 1:2, and 50 nL mixtures were unilaterally delivered (10 nL/min) into the lower thoracic IML (middle‐lateral: −0.35 mm, dorsal‐ventral: −0.5 mm). These two viruses encode the receptor that allows rabies virus (RV) to enter Cre‐expressing neurons and G for transsynaptic transfer. The glass pipette was left in place for 10 min after infusion and slowly removed to avoid backflow. The surgical site was layered sutured and disinfected. Mice were warmed until recovery after subcutaneous injections of Carprofen (5 mg/kg) with sterile saline. Two weeks later, CVS‐EnVA‐ΔG‐EGFP (60 nL) was injected under the same process. One week after spread of the RV, the mice were sacrificed for immunostaining.

### 2.3 Histology

Mice were deeply anesthetized with isoflurane and perfused with 10 mL 0.9% saline followed by 100 mL of 4% paraformaldehyde in 0.1 M phosphate buffer (PB, pH 7.4, 4°C). Brains and lower thoracic spinal segments were removed and placed in 4% paraformaldehyde for 6 h post‐fixation, followed by cryoprotection in 30% sucrose (w/v) in 0.1 M PB until they sank. Tissue was embedded in optimal cutting temperature compound (Epredia), and cryosectioned coronally at 30 μm on a cryostat (Leica CM1950) in three series and were preserved in 0.01 M phosphate‐buffered saline (PBS, pH 7.4). Every one‐third section was mounted using DAPI Fluoromount‐G (Southern Biotech, 0100–20) and coverslipped for imaging.

To confirm the start cells in the IML after virus injection, immunohistochemistry was performed. Spinal segments were incubated with goat anti‐ChAT primary antibody (1:500, catalog number: AB144, Millipore, RRID: AB_11212924) diluted in blocking buffer (PBS with 5% normal donkey serum and 0.25% Triton X‐100) overnight at 4°C. After washing three times in PBS, the sections were incubated with Alexa Fluor‐conjugated IgG antibody (1:1000, catalog number: A‐21447, ThermoFisher) at room temperature for 2 h. Finally, the stained sections were coverslipped with DAPI Fluoromount‐G mounting medium.

### 2.4 Imaging and Data Analysis

Images were captured using a 10 × objective on the VS120 virtual microscopy slide scanning system (VS120, Olympus, Japan), and a 60 × objective by a laser confocal microscope (FV3000, Olympus, Japan). The brain slices were opened in ImageJ and converted from RGB to grayscale. A consistent intensity threshold was applied to visualize labeled neurons, and only signals displaying a clear somatic profile were considered for counting, while diffuse background signals were excluded. Brain region boundaries were defined by matching with the mouse brain atlas [23] and registering each section to the corresponding atlas level using the BigWarp plugin [24] based on the special anatomical structure of the brain. The boundaries of specific brain areas were manually delineated for each section using the “irregular area of interest” tool in ImageJ. The number and density of neuronal bodies were then quantified semiautomatically using the particle analysis function. Cell counts were performed for each animal independently. To ensure reliability, all counts and regional delineations were independently verified by two investigators. The proportion of inputs was calculated for each animal as the ratio of the number of EGFP‐labeled cells in each nucleus to the total number of EGFP‐labeled ones across the whole brain, reflecting the relative contribution of each region to the overall input population. The density of inputs was calculated for each animal as the number of labeled cells within a given brain region divided by the measured area of that region (cells/mm^2^), representing the surface‐normalized concentration of projecting neurons within that structure. Adobe Illustrator was employed to achieve further visualization of whole‐brain projections. All data were processed using GraphPad Prism 9.5 and presented as mean ± SEM. Given the sample size, these quantitative analyses are intended to be descriptive, providing a foundational overview of the input distribution.

## 3 Results

### 3.1 Identification of Monosynaptic Inputs to Lower Thoracic IML Cholinergic Neurons Using a Rabies‐Based Tracing System

**FIGURE 1:**
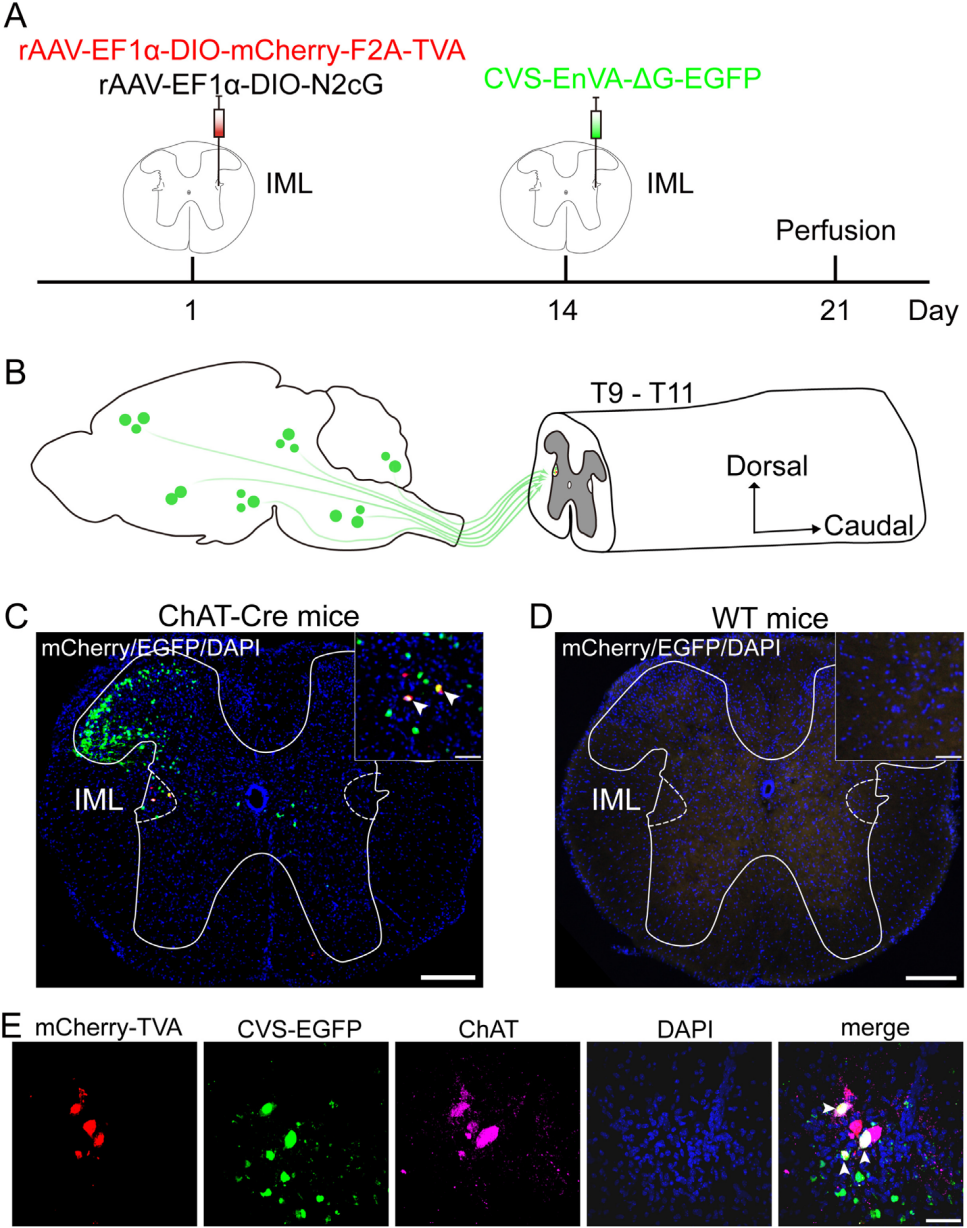
Experimental strategies for tracing monosynaptic inputs to lower thoracic IML cholinergic neurons. (A) Schematic of experimental design. (B) Schematic of the starter cells in the IML and their presynaptic inputs. (C, D) Representative images showing starter cells (yellow) in ChAT‐Cre mice (C), but no labeled neurons in wild‐type mice (D). Scale bars, 200 μm (main image) and 50 μm (inset). (E) Example fluorescence images show starter cells (marked with white arrowheads), which colocalize mCherry, EGFP and ChAT immunoreactive signals in the IML. Scale bar, 40 μm.

To identify the monosynaptic afferent inputs specifically targeting IML cholinergic neurons, we employed a cell‐type‐specific retrograde tracing strategy in ChAT‐Cre mice (Figure 1A). This system was designed to selectively label starter cells in the IML with both helper and RV, enabling the transsynaptic retrograde transport of EGFP to identify monosynaptic inputs (Figure 1A,B). This strategy relies on EnVA‐pseudotyped and glycoprotein (G)‐deficient RV from the CVS‐N2c strain, which is effective at long‐distance transsynaptic transfer and proved essential to efficiently mapping inputs from the brain to the spinal cord [18, 25].

To validate the specificity of this viral targeting strategy, we compared the viral expression between ChAT‐Cre and wild‐type mice. Representative images demonstrated that following the injection, neurons co‐expressing mCherry and EGFP signals were observed in the IML of ChAT‐Cre mice (Figure 1C), while no fluorescent signal was detected in that of wild‐type mice subjected to the same procedure (Figure 1D), confirming the strict Cre‐dependence of our tracing system. The extent of viral expression was confined to T9–T11 segments (Figure S1). Immunostaining further confirmed that these starter cells overlapped with the ChAT immunoreactive signal, verifying their identity as IML cholinergic neurons (Figure 1E). Consequently, this strategy provides a reliable method to visualize monosynaptic afferent inputs to IML cholinergic neurons from the whole brain.

### 3.2 Whole‐Brain Inputs to Lower Thoracic IML Cholinergic Neurons

We next obtained a series of images in the coronal plane throughout the brain to investigate the distribution of monosynaptic inputs to lower thoracic IML cholinergic neurons. The viral tracing strategy previously outlined was employed to identify EGFP‐labeled neurons in the brain, thereby identifying them as presynaptic inputs to cholinergic neurons.

**FIGURE 2:**
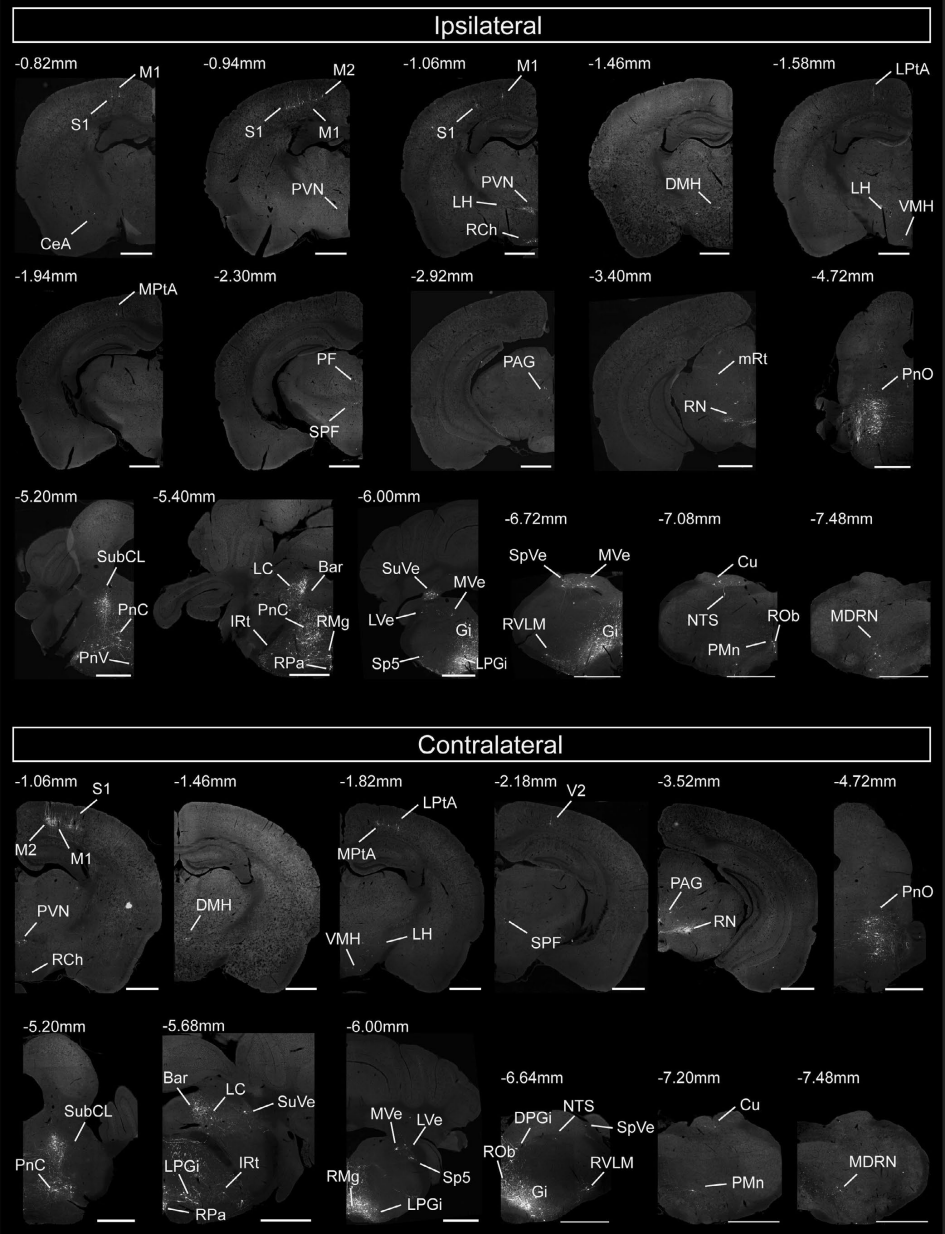
Overviews of labeling retrograde monosynaptic inputs to lower thoracic IML cholinergic neurons from both the ipsilateral and contralateral hemisphere. Scale bar, 1000 μm.

**FIGURE 3:**
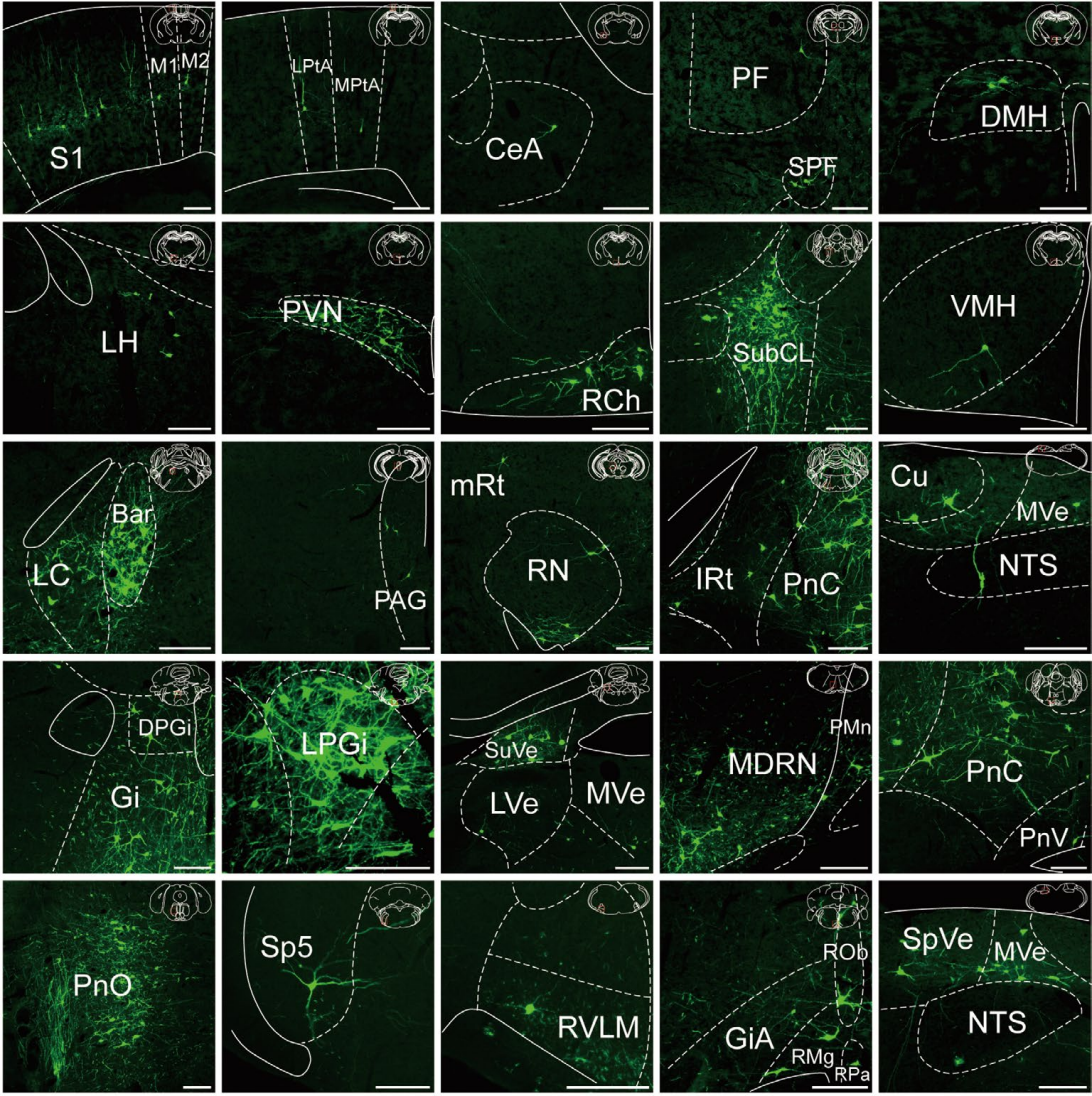
Representative images of nuclei with monosynaptic inputs to lower thoracic IML cholinergic neurons from the ipsilateral hemisphere. Scale bar, 200 μm.

**FIGURE 4:**
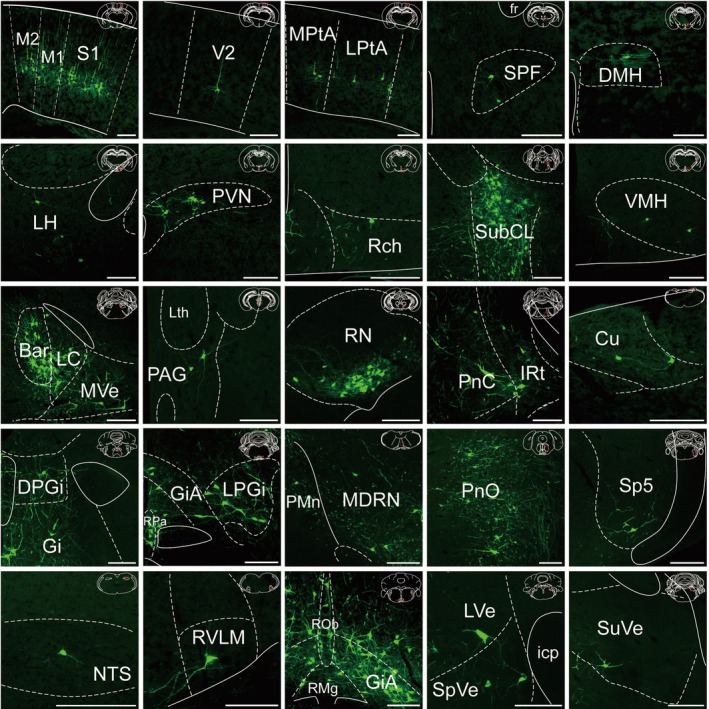
Representative images of nuclei with monosynaptic inputs to lower thoracic IML cholinergic neurons from the contralateral hemisphere. Scale bar, 200 μm.

At the macroscopic level, the results revealed a widespread supraspinal network with presynaptic neurons distributed across 40 discrete brain regions spanning the telencephalon, diencephalon, and brainstem (Figure 2, Table 1). High‐magnification imaging (Figures 3, 4) further visualized distinct anatomical subdivisions, encompassing both established autonomic and homeostatic centers as well as somatic motor and sensory regions.

**TABLE 1:** Abbreviations and classifications of brain structures.

Abbreviation	Name	Brain region	No.
Bar	Barrington's nucleus	Pons	1
CeA	Central amygdaloid nucleus	Telencephalon	2
Cu	Cuneate nucleus	Medulla	3
DMH	Dorsomedial hypothalamic nucleus	Diencephalon	4
DPGi	Dorsal paragigantocellular nucleus	Medulla	5
Gi	Digantocellular reticular nucleus	Medulla	6
IRt	Intermediate reticular nucleus	Medulla	7
LC	Locus coeruleus	Pons	8
LH	Lateral hypothalamic area	Diencephalon	9
LPGi	Lateral paragigantocellular nucleus	Medulla	10
LPtA	Lateral parietal association cortex	Telencephalon	11
LVe	Lateral vestibular nucleus	Medulla	12
M1	Primary motor cortex	Telencephalon	13
M2	Secondary motor cortex	Telencephalon	14
MDRN	Medullary reticular nucleus	Medulla	15
MPtA	Medial parietal association cortex	Telencephalon	16
mRt	Mesencephalic reticular formation	Midbrain	17
MVe	Medial vestibular nucleus	Medulla	18
NTS	Solitary nucleus	Medulla	19
PAG	Periaqueductal gray	Midbrain	20
PF	Parafascicular thalamic nucleus	Diencephalon	21
PMn	Paramedian reticular nucleus	Medulla	22
PnC	Pontine reticular nucleus, caudal part	Pons	23
PnO	Pontine reticular nucleus, oral part	Pons	24
PnV	Pontine reticular nucleus, ventral part	Pons	25
PVN	Paraventricular hypothalamic nucleus	Diencephalon	26
RCh	Retrochiasmatic area	Diencephalon	27
RMg	Raphe magnus nucleus	Medulla	28
RN	Red nucleus	Midbrain	29
ROb	Raphe obscurus nucleus	Medulla	30
RPa	Raphe pallidus nucleus	Medulla	31
RVLM	Rostroventrolateral reticular nucleus	Medulla	32
S1	Primary somatosensory cortex	Telencephalon	33
Sp5	Spinal trigeminal nucleus	Medulla	34
SPF	Subparafascicular thalamic nucleus	Diencephalon	35
SpVe	Spinal vestibular nucleus	Medulla	36
SubCL	Subcoeruleus nucleus	Pons	37
SuVe	Superior vestibular nucleus	Medulla	38
V2	Secondary visual cortex	Telencephalon	39
VMH	Ventromedial hypothalamic nucleus	Diencephalon	40

In the telencephalon, representative images showed monosynaptic inputs from the primary and secondary motor cortices (M1, M2), primary somatosensory cortex (S1), and secondary visual cortex (V2). Robust labeling in diencephalic regions included the LH, PVN, and retrochiasmatic area (RCh). Caudally, the brainstem harbored the most extensive projections. In the midbrain and pons, dense clusters were distinct in the red nucleus (RN), periaqueductal gray (PAG), Barrington's nucleus (Bar), and locus coeruleus (LC). Further caudally, the medulla contained the heaviest concentration of inputs. Particularly intense signals were displayed by the raphe nuclei (RPa, ROb, RMg) and gigantocellular reticular nucleus (Gi), whereas clear labeling was also evident in the RVLM, vestibular nuclei (LVe, MVe), and cuneate nucleus (Cu).

### 3.3 Quantitative Characterization of Input Distribution

**FIGURE 5:**
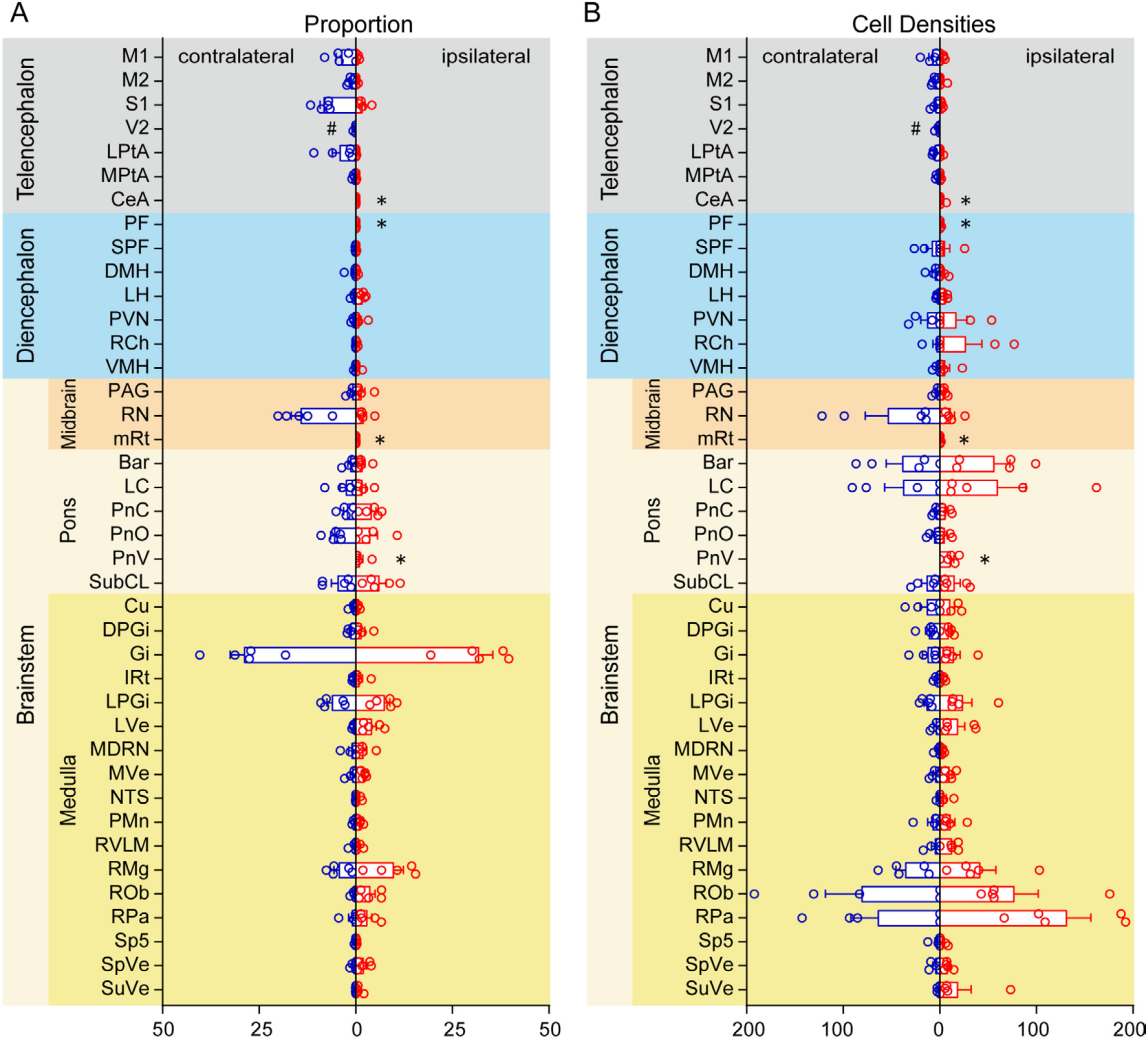
Statistical analysis of bihemispheric projecting lower thoracic IML cholinergic neurons. The proportion (% of total inputs) (A) and cell densities (cells/mm^2^) (B) of five general brain structures inputs to IML: Telencephalon; diencephalon; midbrain, pons, and medulla in the brainstem. Data are represented by mean ± SEM (*n* = 5 mice). # indicates expression observed only in the contralateral side; * indicates expression observed only in the ipsilateral side.

**FIGURE 6:**
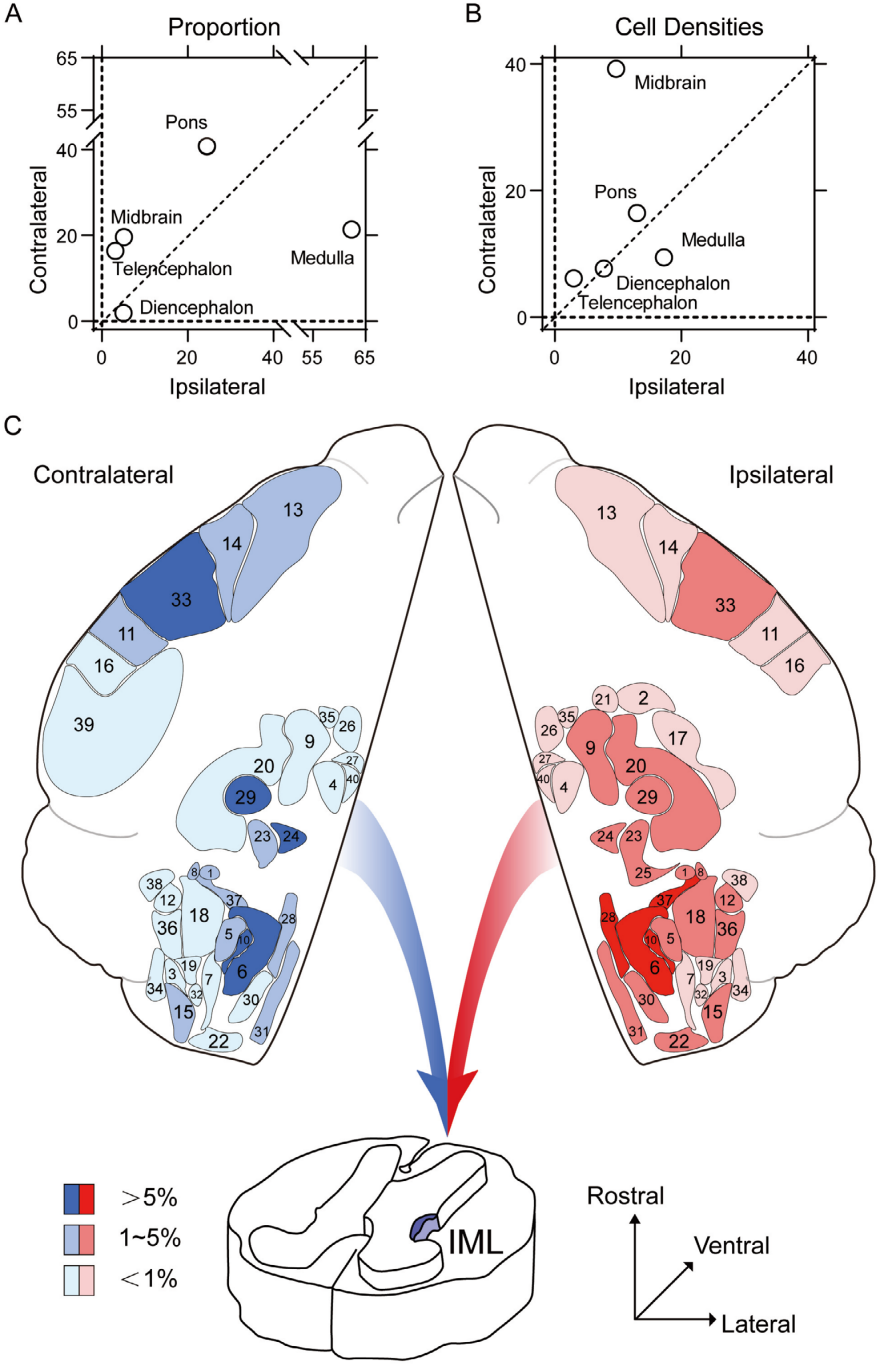
The ipsilateral and contralateral afferent inputs to IML cholinergic neurons. The proportion (% of total inputs) (A) and cell densities (cells/mm^2^) (B) of inputs to the IML from five general brain structures: Telencephalon; diencephalon; midbrain, pons, and medulla in the brainstem. (C) Schematic diagrams presenting the afferent inputs to IML cholinergic neurons on sagittal sections. Refer to Table 1 for the brain region names corresponding to the indicated numerals. Color scale represents the percentage of inputs.

To characterize the hierarchy of these inputs, we quantified the relative contribution of each projecting region (Figure 5). While the brainstem constituted the primary source of afferents bilaterally (Ipsilateral: 91.92%; Contralateral: 81.77%) with prominent labeling encompassing 26 regions, specific regional proportions exhibited observable variations between the hemispheres (Figure 6A,B). We generated comparative scatter plots across five major brain divisions to highlight regions showing descriptive differences in both input proportion and cell density (Figure S2).

On the ipsilateral side, inputs were heavily anchored in the medulla. The Gi emerged as the most prominent source, accounting for 31.86% of the total ipsilateral count. This was accompanied by substantial inputs from other medullary structures, most notably the RMg (9.83%) and lateral paragigantocellular nucleus (LPGi, 7.52%), which together with widespread reticular projections formed the core of the ipsilateral network. In the contralateral hemisphere, the Gi remained the primary source (28.95%), underscoring its anatomical dominance on both sides. However, the distribution pattern shifted rostrally with the emergence of midbrain inputs. Most notably, the RN became a major contributor (14.29%) in the contralateral hemisphere, compared to its ipsilateral proportion (2.02%). A similar contralateral bias was evident in the telencephalon. S1 (8.35%) and M1 (3.80%) provided significant inputs, whereas their ipsilateral contributions were negligible (< 1%).

When normalized for region size, specific autonomic centers exhibited high local concentrations (> 5 cells/mm^2^) despite varying total numbers. The raphe nuclei displayed the highest density bilaterally, with the RPa reaching 131.53 cells/mm^2^ ipsilaterally and the ROb reaching 81.28 cells/mm^2^ contralaterally. Notably, the RVLM, despite contributing a minor proportion to the total count (< 1%), exhibited a high cell density (12.41 cells/mm^2^), reflecting a compact but potent projection.

To provide a comprehensive visual summary of these bihemispheric inputs, we mapped the afferent distributions of IML cholinergic neurons and displayed the proportions (Figure 6C, brain regions are numerically coded as listed in Table 1). These observed patterns suggest that the lower thoracic IML receives broad monosynaptic inputs from widespread supraspinal regions, suggesting its potential to serve as an integration hub for linking central neural networks directly to sympathetic outflow.

## 4 Discussion

The IML receives extensive descending inputs from supraspinal regions, and the integration and coordination of these inputs can result in the generation of final central sympathetic signals for different organs. In particular, the lower thoracic segment is critical for splanchnic sympathetic outflow to key abdominal viscera, including the adrenal medulla, liver, pancreas, and kidneys, thereby regulating metabolic homeostasis. Given this role, we mapped whole‐brain inputs to cholinergic neurons in the lower thoracic IML. These neurons receive extensive afferent projections from widespread nuclei of the telencephalon, diencephalon, and brainstem, and these innervations are generally organized in a bilateral manner. These findings provide an anatomical blueprint of the upstream networks controlling lower thoracic IML and pave the way for understanding brain‐spinal cord pathway functions in specific physiological contexts.

### 4.1 Global Assessment of Supraspinal Inputs to Lower Thoracic IML

Given the critical role of the IML in mediating sympathetic outflow from the brain to the periphery to coordinate autonomic responses, early studies utilized retrograde tracers such as horseradish peroxidase or fluorescent tracers to delineate its supraspinal connectivity [12, 15, 26]. These investigations identified key input sources projecting to the lower thoracic IML, including the dorsomedial hypothalamic nucleus (DMH), LH, RVLM, solitary nucleus (NTS), and PVN. Furthermore, PRV tracing demonstrated that the RVLM, PVN, and raphe nuclei were among the first regions infected following injection into the adrenal gland, suggesting a potential projection relationship with sympathetic preganglionic neurons in lower thoracic segments [27]. Another study combining anterograde tracing with immunofluorescence confirmed direct projections from the NTS to these cholinergic neurons [28]. Consistent with these findings, we observed robust labeling in these canonical autonomic centers, supporting the reliability of our results.

However, the complete supraspinal landscape governing lower thoracic IML cholinergic neurons remains to be fully elucidated due to the inherent limitations of previous approaches. Traditional tracers are prone to local diffusion, compromising anatomical precision, and lack cell‐type specificity, inevitably labeling non‐cholinergic interneurons within the IML [29]. Regarding the PRV tracing strategy, the nature of the virus makes it difficult to distinguish direct monosynaptic inputs from upstream indirect connections. To overcome these limitations, we employed the CVS‐N2c rabies virus strain. As demonstrated by Chapman et al. [30], this enhanced efficiency enables reliable labeling even when the number of starter cells is limited, which is a critical advantage when targeting sparse populations such as spinal preganglionic neurons. Thus, the projection map obtained in our study can represent a comprehensive panorama of the supraspinal network governing IML cholinergic neurons.

We identified direct monosynaptic inputs from the cerebral cortices (M1, M2, S1) to the lower thoracic IML, a pathway previously thought to be sparse based on limited evidence [31]. While PRV injections into the stomach [32] and adrenal medulla [33] only hinted at cortical participation in the sympathetic network, our results provide strong anatomical evidence supporting a direct corticospinal pathway targeting sympathetic preganglionic neurons. This structural substrate may underlie rapid cortical modulation of sympathetic outflow, coordinating somatomotor and autonomic adjustments during complex behavioral contexts. Additionally, the high sensitivity of our viral strategy revealed several previously unidentified input regions, including the V2, Cu, and parietal association cortices (MPtA, LPtA). Given their known involvement in visual processing [34], proprioception [35], and sensorimotor integration [36], these projections suggest a potential mechanism for integrating sensory cues directly with sympathetic outflow, allowing for rapid autonomic adjustments in response to external stimuli.

Despite the specific cortical inputs, our quantitative analysis reveals that the supraspinal network regulating the lower thoracic IML is predominantly weighted towards the brainstem. This aligns with the segmental specificity described by Wang et al. [37], where the thoracic spinal cord is dominated by brainstem innervation. Within this network, we identified the Gi as the most abundant source of inputs, which is classically characterized as a somatic motor center driving locomotion [38]. Recent studies have highlighted brain regions capable of coupling somatic movement and sympathetic output, among which Gi has been shown to have this capacity by projecting to the anterior horn and the IML [39]. Our identification of dense Gi projections to lower thoracic IML cholinergic neurons provides direct anatomical evidence suggesting the Gi as a putative node in parallel somatomotor and sympathetic circuits.

This study not only provides a reliable anatomical mapping of the neural architecture governing lower thoracic IML cholinergic neurons but also offers a structural framework for generating hypotheses regarding their potential roles in metabolic homeostasis and somato‐autonomic coupling.

### 4.2 Implications for the Lower Thoracic IML Involvement in Homeostatic Regulation

The regulation of energy metabolism represents a complex mechanism where maintaining homeostasis is fundamental to survival. The sympathetic nervous system plays an indispensable role in this process. Distinct from the upper thoracic segments that predominantly govern cardiovascular function, the lower thoracic IML serves as a specialized output channel for the homeostasis of abdominal viscera [5]. Anatomically, this region functions as the direct preganglionic source for the celiac and superior mesenteric ganglia, the key autonomic relays governing abdominal organs [4, 10]. Through this neural architecture, lower thoracic IML cholinergic neurons modulate essential metabolic organs, including the pancreas, liver, and adipose tissue [40, 41]. Our tracing results identify that the lower thoracic IML receives widespread, direct inputs from multiple brain regions, highlighting the anatomical complexity of its integrative function and supporting the characterization of this region as a potential convergence hub for diverse homeostatic signals.

Among diverse homeostatic functions, glucose metabolism has emerged as a particularly well‐characterized example of central regulation of peripheral physiology mediated by lower thoracic IML cholinergic neurons. It is now established that the brain modulates hepatic glycogenolysis and gluconeogenesis through this specific spinal segment to adapt to dynamic glycemic demands [42]. Injection of triiodothyronine into the PVN to mimic a high metabolic demand was found to drive hepatic glucose production, a regulatory effect demonstrated to be IML‐dependent through hepatic sympathetic denervation [43]. Furthermore, viral tracing and optogenetic studies have delineated a polysynaptic circuit from the PVN via the VLM to the IML that promotes hepatic glycogenolysis under stress [14]. Regarding direct pathways, monosynaptic PVN projections to the lower thoracic IML have been shown to modulate pancreatic β‐cells, exerting rapid inhibition of insulin secretion during hypoglycemia [44]. Our current findings corroborate and extend these observations. We observed dense labeling in the PVN, providing a robust anatomical basis for its projections to lower thoracic IML cholinergic neurons. While consistent with previous chemical tracing studies that reported ipsilateral dominance, our viral tracing revealed a more balanced bilateral distribution [45]. This observed distribution pattern likely arises from the superior sensitivity of the viral tracing method, allowing contralateral projections to be adequately labeled. Unlike spinal ventral horn motor neurons that predominantly receive contralateral innervation for lateralized voluntary movement, the lower thoracic IML is primarily engaged in systemic homeostatic regulation rather than lateralized visceral control. We hypothesize that this bilateral organization confers functional redundancy in the regulation of sympathetic tone, ensuring the resilience of autonomic responses to vital organs [46, 47].

Our findings also revealed direct connections from regions traditionally associated with other autonomic functions. The RCh and Bar, regions known to regulate sympathetic tone [48] and micturition [49], were previously thought to connect to the IML indirectly via downstream nuclei like the RVLM or PAG. However, our monosynaptic tracing confirms the existence of direct connections, challenging this assumption. These pathways likely coordinate the necessary splanchnic vascular or metabolic adjustments during complex autonomic behaviors.

By bridging the central inputs mapped in our study with specific downstream targets, lower thoracic IML is positioned as a putative integrative hub for the central regulation of peripheral physiology. The diverse array of inputs positions the lower thoracic IML as a versatile coordinator of multiple homeostatic processes beyond glucose regulation alone. Identifying these inputs establishes a critical anatomical foundation for future experimental investigations into the central neural regulation of metabolism.

### 4.3 Implications for the Lower Thoracic IML Involvement in Coupling of Motor Control and Sympathetic Outflow

Our study reveals direct supraspinal inputs to lower thoracic IML cholinergic neurons, originating from brain regions classically associated with somatic motor control. These include the contralateral RN, the source of the rubrospinal tract [50, 51]; corticospinal tracts conveying goal‐directed commands; and specific brainstem reticular formations—namely the Gi, LPGi, and medullary reticular nucleus (MDRN) identified via retrograde tracing of spinal V1 interneurons [30]. The close alignment of these inputs with well‐established motor pathways not only validates the reliability of our tracing data but, crucially, demonstrates that the IML is structurally integrated into the broader motor control network.

The IML is the established final integration center for sympathetic signals. Therefore, these findings of direct inputs from motor regions provide a solid anatomical foundation for the physiological coupling of motor and sympathetic functions. This coupling is essential for the execution of diverse behavioral states, from the synchronization of skeletal muscles and sympathetic execution (e.g., increased heart rate and blood pressure) during running or the fight‐or‐flight response [52], to the concurrent muscle atonia and suppressed sympathetic tone during rapid eye movement sleep [53, 54]. We therefore propose that these supraspinal inputs from motor‐related regions are fundamental for orchestrating the precise coordination of somatic motor and sympathetic outflow.

However, the mechanism underlying this motor‐sympathetic coupling is more complex than a simple “on/off” correspondence. Elaborate motor behaviors (such as stopping, turning, and accelerating [55]) necessitate finer sympathetic control patterns. This functional complexity likely stems from neuronal heterogeneity within the IML itself, where distinct subpopulations may separately and in parallel regulate somatic motor tone and sympathetic activity [56].

## 5 Limitations

We defined T9‐T11 as a unified functional domain in the present study, while acknowledging that the IML forms a longitudinal sympathetic column extending from T1 to L2. Previous studies suggested that supraspinal neurons projecting to different segments are largely intermixed, without clear clustering or topographic organization according to spinal level [12]. Therefore, potential differences either among individual segments within the lower thoracic IML or across distinct IML domains require more detailed and refined experiments. Regarding the methodology, although we employed an advanced viral tracing strategy, the inherent limitations of RV tracing must be considered. The efficiency exhibits significant heterogeneity across brain regions [57], and specific synaptic structural features may physically or biochemically impede effective viral transmission [58]. As an anatomical method, RV tracing cannot directly infer synaptic connection strength or distinguish between physiological types (e.g., excitatory or inhibitory). Given that the transsynaptic spread process does not necessarily cross all synapses with equal probability [59], the observed labeling density does not necessarily reflect functional connection efficacy [30]. Subsequent functional experiments are essential to validate the physiological significance and regulatory roles of these anatomical pathways.

In summary, this study proposes the IML not only as an origin of sympathetic output, but as a potential higher‐order coordination center that integrates descending motor commands with homeostatic demands. This structural framework suggests a pathway through which central motor intentions could be integrated into appropriately tuned peripheral autonomic responses, thereby serving as a key node in the brain–body axis for behavioral regulation. Future work should aim to delineate the functional neuronal subtypes within the lower thoracic IML and elucidate the molecular and circuit mechanisms by which distinct brain–spinal pathways synergistically coordinate the interactions between the motor and sympathetic systems.

## 6 Conclusion

This study maps the monosynaptic inputs to cholinergic neurons in the lower thoracic IML, highlighting their potential role as a convergent hub for integrating somatic motor and homeostatic signals. Our findings outline the anatomical substrate potentially underlying the coupling of central motor commands with sympathetic outflow and brain–body axis coordination. Functional dissection of these identified pathways is further needed to understand their contribution to pathological states such as autonomic neuropathy, metabolic disorders, and spinal cord injury.

## Author Contributions

**Yuan‐jun Yang:** methodology, investigation, gormal analysis, fata vuration, visualization, writing – original draft, writing – review and editing. **Kai‐ying Zhang:** conceptualization, methodology, writing – original draft, supervision, formal analysis. **Bin‐bin Li:** conceptualization, methodology, visualization. **Xia‐wan Liu:** methodology, visualization. **Jing‐rong Li:** methodology, visualization. **Yi‐nuo Liu:** visualization. **Yu‐han Liu:** visualization. **Ji Li:** validation, writing – review and editing. **Xiang‐shan Yuan:** validation, methodology, visualization, writing – original draft, writing – review and editing, funding acquisition. **Ming Zhong:** conceptualization, methodology, validation, writing – original draft, writing – review and editing, supervision, project administration, funding acquisition.

## Funding

This work was supported by Shanghai Key Laboratory of Lung Inflammation and Injury. Open Project of State Key Laboratory of Respiratory Disease (SKLRD‐ОР‐202321). Key Disciplines of the Three‐year Action Plan for Strengthening the Construction of Public Health System in Shanghai (GWVI‐11.1‐14). National Natural Science Foundation of China (32371192, 32171142). Open Project of Key Laboratory of Longevity and Aging‐related Diseases (Guangxi Medical University), Ministry of Education (KLLAD202501).

## Ethics Statement

Animal experiments were conducted in accordance with the Committee on the Ethics of Animal Experiments of Fudan University Shanghai Medical College (Permit No. 20190221–028).

## Conflicts of Interest

The authors declare no conflicts of interest.

## Supporting information


**Figure S1:** The accuracy of the viral injection locations within the T9‐T11 IML. (A) Schematic of the starter cells in the T9‐T11 segments. (B‐F) Representative images from T8 to T12 segments in ChAT‐Cre mice.
**Figure S2:** Descriptive comparison of hemispheric distribution in supraspinal inputs to IML cholinergic neurons. (A‐E) Comparative scatter plots illustrating the proportion of total inputs originating from the ipsilateral versus contralateral sides for individual nuclei. Data are segmented by major brain divisions: Telencephalon (A), Diencephalon (B), Midbrain (C), Pons (D), and Medulla (E). Each data point represents a specific brain region. The diagonal line serves as a reference for bilateral symmetry. (F) Legend for panels A‐E. The visual style of the data points categorizes the magnitude of the inter‐hemispheric difference in projection proportion: empty circles represent a difference of 0%–1%, light‐colored circles represent 1%–5%, and dark‐colored circles represent > 5%. (G‐K) Comparative scatter plots illustrating the cell density (cells/mm^2^) of afferent inputs from the ipsilateral versus contralateral sides. Panels correspond to the Telencephalon (G), Diencephalon (H), Midbrain (I), Pons (J), and Medulla (K). (L) Legend for panels G‐K, and the circle styles indicate the magnitude of the inter‐hemispheric difference in cell density. Points deviating from the diagonal line highlight nuclei with observable hemispheric variation in either proportion or density.

## Data Availability

The data that support the findings of this study are available from the corresponding author upon reasonable request.

## References

[1] N. Yapici , “The Ancient Dialogue Between Brain and Body,” Current Biology 35 (2025): R963–r967. 41118739 10.1016/j.cub.2025.08.069 PMC13452438

[2] S. Aten , N. Lynch , C. B. Saper , and N. L. S. Machado , “A Brain‐Body Perspective on Thermoregulatory Adaptation,” Current Biology 35 (2025): R1016–r1028. 41118728 10.1016/j.cub.2025.09.023 PMC12614449

[3] M. Sammons , M. C. Popescu , J. Chi , S. D. Liberles , N. Gogolla , and A. Rolls , “Brain‐Body Physiology: Local, Reflex, and Central Communication,” Cell 187 (2024): 5877–5890. 39423806 10.1016/j.cell.2024.08.050 PMC11624509

[4] T. Wang , B. Teng , D. R. Yao , W. Gao , and Y. Oka , “Organ‐Specific Sympathetic Innervation Defines Visceral Functions,” Nature 637 (2025): 895–902. 39604732 10.1038/s41586-024-08269-0

[5] E. A. Wehrwein , H. S. Orer , and S. M. Barman , “Overview of the Anatomy, Physiology, and Pharmacology of the Autonomic Nervous System,” Compr Physiol 6 (2016): 1239–1278. 27347892 10.1002/cphy.c150037

[6] R. P. Barber , P. E. Phelps , C. R. Houser , G. D. Crawford , P. M. Salvaterra , and J. E. Vaughn , “The Morphology and Distribution of Neurons Containing Choline Acetyltransferase in the Adult Rat Spinal Cord: An Immunocytochemical Study,” Journal of Comparative Neurology 229 (1984): 329–346. 6389613 10.1002/cne.902290305

[7] E. Scott‐Solomon , E. Boehm , and R. Kuruvilla , “The Sympathetic Nervous System in Development and Disease,” Nature Reviews Neuroscience 22 (2021): 685–702. 34599308 10.1038/s41583-021-00523-y PMC8530968

[8] M. M. Moura , A. Monteiro , A. J. Salgado , N. A. Silva , and S. Monteiro , “Disrupted Autonomic Pathways in Spinal Cord Injury: Implications for the Immune Regulation,” Neurobiology of Disease 195 (2024): 106500. 38614275 10.1016/j.nbd.2024.106500

[9] M. R. Alkaslasi , Z. E. Piccus , S. Hareendran , et al., “Single Nucleus RNA‐Sequencing Defines Unexpected Diversity of Cholinergic Neuron Types in the Adult Mouse Spinal Cord,” Nature Communications 12 (2021): 2471. 10.1038/s41467-021-22691-2 PMC8087807 33931636

[10] Y. Harima , M. Tsurutani , S. Yamada , et al., “Parallel Labeled‐Line Organization of Sympathetic Outflow for Selective Organ Regulation in Mice,” Nature Communications 15 (2024): 10478. 10.1038/s41467-024-54928-1 PMC11631959 39658565

[11] M. C. Viñuela and P. J. Larsen , “Identification of NPY‐Induced c‐Fos Expression in Hypothalamic Neurones Projecting to the Dorsal Vagal Complex and the Lower Thoracic Spinal Cord,” Journal of Comparative Neurology 438 (2001): 286–299. 11550173 10.1002/cne.1316

[12] D. C. Tucker and C. B. Saper , “Specificity of Spinal Projections From Hypothalamic and Brainstem Areas Which Innervate Sympathetic Preganglionic Neurons,” Brain Research 360 (1985): 159–164. 4075168 10.1016/0006-8993(85)91231-4

[13] T. Shahar and M. Palkovits , “Cross Over of Forebrain and Brainstem Neuronal Projections to Spinal Cord Sympathetic Preganglionic Neurons in the Rat,” Stress 10 (2007): 145–152. 17514583 10.1080/10253890701424712

[14] Z. Zhao , L. Wang , W. Gao , et al., “A Central Catecholaminergic Circuit Controls Blood Glucose Levels During Stress,” Neuron 95 (2017): 138–152.e135. 28625488 10.1016/j.neuron.2017.05.031

[15] S. A. Deuchars and V. K. Lall , “Sympathetic Preganglionic Neurons: Properties and Inputs,” Compr Physiol 5 (2015): 829–869. 25880515 10.1002/cphy.c140020

[16] E. M. Callaway and L. Luo , “Monosynaptic Circuit Tracing With Glycoprotein‐Deleted Rabies Viruses,” Journal of Neuroscience 35 (2015): 8979–8985. 26085623 10.1523/JNEUROSCI.0409-15.2015 PMC4469731

[17] A. C. Bostan , R. P. Dum , and P. L. Strick , “The Basal Ganglia Communicate With the Cerebellum,” Proceedings of the National Academy of Sciences of the United States of America 107 (2010): 8452–8456. 20404184 10.1073/pnas.1000496107 PMC2889518

[18] T. R. Reardon , A. J. Murray , G. F. Turi , et al., “Rabies Virus CVS‐N2c(ΔG) Strain Enhances Retrograde Synaptic Transfer and Neuronal Viability,” Neuron 89 (2016): 711–724. 26804990 10.1016/j.neuron.2016.01.004 PMC4760870

[19] K. Haenraets , G. W. Albisetti , E. Foster , and H. Wildner , “Adeno‐Associated Virus‐Mediated Transgene Expression in Genetically Defined Neurons of the Spinal Cord,” Journal of Visualized Experiments (2018): 57382. 29806830 10.3791/57382 PMC6101182

[20] C. Bellardita , M. Marcantoni , P. Löw , and O. Kiehn , “Sacral Spinal Cord Transection and Isolated Sacral Cord Preparation to Study Chronic Spinal Cord Injury in Adult Mice,” Bio‐Protocol 8 (2018): e2784. 29795778 10.21769/BioProtoc.2784 PMC5961933

[21] M. J. Cook , The Anatomy of the Laboratory Mouse (Academic Press, 1965).

[22] M. Harrison , A. O'Brien , L. Adams , et al., “Vertebral Landmarks for the Identification of Spinal Cord Segments in the Mouse,” Neuroimage 68 (2013): 22–29. 23246856 10.1016/j.neuroimage.2012.11.048

[23] K. Franklin and G. Paxinos , Paxinos and Franklin's the Mouse Brain in Stereotaxic Coordinates, 5th Edition ed. (Elsevier Academic Press, 2019).

[24] J. A. Bogovic , P. Hanslovsky , A. Wong , and S. Saalfeld , “Robust Registration of Calcium Images by Learned Contrast Synthesis,” Paper Presented at: 2016 IEEE 13th International Symposium on Biomedical Imaging (ISBI) (2016): 13–16.

[25] H. G. Kuypers and V. A. Maisky , “Retrograde Axonal Transport of Horseradish Peroxidase From Spinal Cord to Brain Stem Cell Groups in the Cat,” Neuroscience Letters 1 (1975): 9–14. 19604744 10.1016/0304-3940(75)90004-x

[26] K. Amendt , J. Czachurski , K. Dembowsky , and H. Seller , “Bulbospinal Projections to the Intermediolateral Cell Column: A Neuroanatomical Study,” Journal of the Autonomic Nervous System 1 (1979): 103–107. 575994 10.1016/0165-1838(79)90009-2

[27] A. M. Strack , W. B. Sawyer , J. H. Hughes , K. B. Platt , and A. D. Loewy , “A General Pattern of CNS Innervation of the Sympathetic Outflow Demonstrated by Transneuronal Pseudorabies Viral Infections,” Brain Research 491 (1989): 156–162. 2569907 10.1016/0006-8993(89)90098-x

[28] I. J. Llewellyn‐Smith , N. Marina , R. N. Manton , F. Reimann , F. M. Gribble , and S. Trapp , “Spinally Projecting Preproglucagon Axons Preferentially Innervate Sympathetic Preganglionic Neurons,” Neuroscience 284 (2015): 872–887. 25450967 10.1016/j.neuroscience.2014.10.043 PMC4300405

[29] Y. Ruska , A. Csibi , B. Dorogházi , et al., “Topography of the GLP‐1/GLP‐1 Receptor System in the Spinal Cord of Male Mice,” Scientific Reports 14 (2024): 14403. 38909126 10.1038/s41598-024-65442-1 PMC11193760

[30] P. D. Chapman , A. S. Kulkarni , A. J. Trevisan , et al., “A Brain‐Wide Map of Descending Inputs Onto Spinal V1 Interneurons,” Neuron 113 (2025): 524–538.e526. 39719703 10.1016/j.neuron.2024.11.019 PMC11842218

[31] K. M. Hurley , H. Herbert , M. M. Moga , and C. B. Saper , “Efferent Projections of the Infralimbic Cortex of the Rat,” Journal of Comparative Neurology 308 (1991): 249–276. 1716270 10.1002/cne.903080210

[32] D. J. Levinthal and P. L. Strick , “Multiple Areas of the Cerebral Cortex Influence the Stomach,” Proceedings of the National Academy of Sciences of the United States of America 117 (2020): 13078–13083. 32434910 10.1073/pnas.2002737117 PMC7293610

[33] R. P. Dum , D. J. Levinthal , and P. L. Strick , “Motor, Cognitive, and Affective Areas of the Cerebral Cortex Influence the Adrenal Medulla,” Proceedings of the National Academy of Sciences of the United States of America 113 (2016): 9922–9927. 27528671 10.1073/pnas.1605044113 PMC5024624

[34] J. H. Siegle , X. Jia , S. Durand , et al., “Survey of Spiking in the Mouse Visual System Reveals Functional Hierarchy,” Nature 592 (2021): 86–92. 33473216 10.1038/s41586-020-03171-x PMC10399640

[35] C. Versteeg , R. H. Chowdhury , and L. E. Miller , “Cuneate Nucleus: The Somatosensory Gateway to the Brain,” Current Opinion in Physiology 20 (2021): 206–215. 33869911 10.1016/j.cophys.2021.02.004 PMC8049169

[36] D. Lyamzin and A. Benucci , “The Mouse Posterior Parietal Cortex: Anatomy and Functions,” Neuroscience Research 140 (2019): 14–22. 30465783 10.1016/j.neures.2018.10.008

[37] Z. Wang , A. Romanski , V. Mehra , et al., “Brain‐Wide Analysis of the Supraspinal Connectome Reveals Anatomical Correlates to Functional Recovery After Spinal Injury,” eLife 11 (2022): e76254. 35838234 10.7554/eLife.76254 PMC9345604

[38] R. Leiras , J. M. Cregg , and O. Kiehn , “Brainstem Circuits for Locomotion,” Annual Review of Neuroscience 45 (2022): 63–85. 10.1146/annurev-neuro-082321-025137 34985919

[39] I. A. Kerman , “Organization of Brain Somatomotor‐Sympathetic Circuits,” Experimental Brain Research 187 (2008): 1–16. 18369609 10.1007/s00221-008-1337-5

[40] W. Ren , M. Hua , F. Cao , and W. Zeng , “The Sympathetic‐Immune Milieu in Metabolic Health and Diseases: Insights From Pancreas, Liver, Intestine, and Adipose Tissues,” Advanced Science (Weinheim) 11 (2024): e2306128. 10.1002/advs.202306128 PMC10885671 38039489

[41] N. Martinez‐Sanchez , O. Sweeney , D. Sidarta‐Oliveira , A. Caron , S. A. Stanley , and A. I. Domingos , “The Sympathetic Nervous System in the 21st Century: Neuroimmune Interactions in Metabolic Homeostasis and Obesity,” Neuron 110 (2022): 3597–3626. 36327900 10.1016/j.neuron.2022.10.017 PMC9986959

[42] A. Zsombok , L. D. Desmoulins , and A. V. Derbenev , “Sympathetic Circuits Regulating Hepatic Glucose Metabolism: Where We Stand,” Physiological Reviews 104 (2024): 85–101. 37440208 10.1152/physrev.00005.2023 PMC11281813

[43] L. P. Klieverik , S. F. Janssen , A. van Riel , et al., “Thyroid Hormone Modulates Glucose Production via a Sympathetic Pathway From the Hypothalamic Paraventricular Nucleus to the Liver,” Proceedings of the National Academy of Sciences of the United States of America 106 (2009): 5966–5971. 19321430 10.1073/pnas.0805355106 PMC2660059

[44] I. Papazoglou , J. H. Lee , Z. Cui , et al., “A Distinct Hypothalamus‐To‐β Cell Circuit Modulates Insulin Secretion,” Cell Metabolism 34 (2022): 285–298.e287. 35108515 10.1016/j.cmet.2021.12.020 PMC8935365

[45] A. D. Shafton , A. Ryan , and E. Badoer , “Neurons in the Hypothalamic Paraventricular Nucleus Send Collaterals to the Spinal Cord and to the Rostral Ventrolateral Medulla in the Rat,” Brain Research 801 (1998): 239–243. 9729407 10.1016/s0006-8993(98)00587-3

[46] A. C. Schapiro , J. L. McClelland , S. R. Welbourne , T. T. Rogers , and M. A. Lambon Ralph , “Why Bilateral Damage Is Worse Than Unilateral Damage to the Brain,” Journal of Cognitive Neuroscience 25 (2013): 2107–2123. 23806177 10.1162/jocn_a_00441

[47] B. E. P. Mizusaki and C. O'Donnell , “Neural Circuit Function Redundancy in Brain Disorders,” Current Opinion in Neurobiology 70 (2021): 74–80. 34416675 10.1016/j.conb.2021.07.008 PMC8694099

[48] E. R. Ribeiro‐Barbosa , A. L. Skorupa , J. Cipolla‐Neto , and N. S. Canteras , “Projections of the Basal Retrochiasmatic Area: A Neural Site Involved in the Photic Control of Pineal Metabolism,” Brain Research 839 (1999): 35–40. 10482796 10.1016/s0006-8993(99)01685-6

[49] R. J. Valentino , L. A. Pavcovich , and H. Hirata , “Evidence for Corticotropin‐Releasing Hormone Projections From Barrington's Nucleus to the Periaqueductal Gray and Dorsal Motor Nucleus of the Vagus in the Rat,” Journal of Comparative Neurology 363 (1995): 402–422. 8847408 10.1002/cne.903630306

[50] H. Liang , G. Paxinos , and C. Watson , “The Red Nucleus and the Rubrospinal Projection in the Mouse,” Brain Structure & Function 217 (2012): 221–232. 21927901 10.1007/s00429-011-0348-3

[51] Y. He , G. Madeo , Y. Liang , et al., “A Red Nucleus‐VTA Glutamate Pathway Underlies Exercise Reward and the Therapeutic Effect of Exercise on Cocaine Use,” Science Advances 8 (2022): eabo1440. 36054363 10.1126/sciadv.abo1440 PMC10848951

[52] L. C. Michelini , D. S. O'Leary , P. B. Raven , and A. C. Nóbrega , “Neural Control of Circulation and Exercise: A Translational Approach Disclosing Interactions Between Central Command, Arterial Baroreflex, and Muscle Metaboreflex,” American Journal of Physiology. Heart and Circulatory Physiology 309 (2015): H381–H392. 26024683 10.1152/ajpheart.00077.2015 PMC4631530

[53] D. Liu and Y. Dan , “A Motor Theory of Sleep‐Wake Control: Arousal‐Action Circuit,” Annual Review of Neuroscience 42 (2019): 27–46. 10.1146/annurev-neuro-080317-061813 30699051

[54] T. E. Scammell , E. Arrigoni , and J. O. Lipton , “Neural Circuitry of Wakefulness and Sleep,” Neuron 93 (2017): 747–765. 28231463 10.1016/j.neuron.2017.01.014 PMC5325713

[55] G. Gatto and M. Goulding , “Locomotion Control: Brainstem Circuits Satisfy the Need for Speed,” Current Biology 28 (2018): R256–r259. 29558639 10.1016/j.cub.2018.01.068 PMC5942195

[56] Z. Zhang , J. Su , J. Tang , et al., “Spinal Projecting Neurons in Rostral Ventromedial Medulla Co‐Regulate Motor and Sympathetic Tone,” Cell 187 (2024): 3427–3444.e3421. 38733990 10.1016/j.cell.2024.04.022 PMC11193620

[57] L. Sun , Y. Tang , K. Yan , et al., “Differences in Neurotropism and Neurotoxicity Among Retrograde Viral Tracers,” Molecular Neurodegeneration 14 (2019): 8. 30736827 10.1186/s13024-019-0308-6 PMC6368820

[58] Z. Li , Z. Chen , G. Fan , A. Li , J. Yuan , and T. Xu , “Cell‐Type‐Specific Afferent Innervation of the Nucleus Accumbens Core and Shell,” Frontiers in Neuroanatomy 12 (2018): 84. 30459564 10.3389/fnana.2018.00084 PMC6232828

[59] A. R. Nectow and E. J. Nestler , “Viral Tools for Neuroscience,” Nature Reviews Neuroscience 21 (2020): 669–681. 33110222 10.1038/s41583-020-00382-z PMC7808553

