# Supplementary material for: Whole‐Brain Neural Connectivity to Cholinergic Neurons in the Lower Thoracic Intermediolateral Column
Source: CNS Neurosci Ther. 2026 Apr 27;32(4):e70902. doi: 10.1002/cns.70902 (PMC13121841; doi:10.1002/cns.70902)
Supplement: Supplementary file 1 — Figure S1: The accuracy of the viral injection locations within the T9‐T11 IML. (A) Schematic of the starter cells in the T9‐T11 segments. (B‐F) Representative images from T8 to T12 segments in ChAT‐Cre mice. Figure S2: Descriptive comparison of hemispheric distribution in supraspinal inputs to IML cholinergic neurons. (A‐E) Comparative scatter plots illustrating the proportion of total inputs originating from the ipsilateral versus contralateral sides for individual nuclei. Data are segmented by major brain divisions: Telencephalon (A), Diencephalon (B), Midbrain (C), Pons (D), and Medulla (E). Each data point represents a specific brain region. The diagonal line serves as a reference for bilateral symmetry. (F) Legend for panels A‐E. The visual style of the data points categorizes the magnitude of the inter‐hemispheric difference in projection proportion: empty circles represent a difference of 0%–1%, light‐colored circles represent 1%–5%, and dark‐colored circles represent > 5%. (G‐K) Comparative scatter plots illustrating the cell density (cells/mm2) of afferent inputs from the ipsilateral versus contralateral sides. Panels correspond to the Telencephalon (G), Diencephalon (H), Midbrain (I), Pons (J), and Medulla (K). (L) Legend for panels G‐K, and the circle styles indicate the magnitude of the inter‐hemispheric difference in cell density. Points deviating from the diagonal line highlight nuclei with observable hemispheric variation in either proportion or density. [file CNS-32-e70902-s001.docx]

**Supplementary Information for**

**Whole-brain Neural Connectivity to Cholinergic Neurons in the Lower Thoracic Intermediolateral Column**

**Yuan-jun Yang**^1,^^2, *^**, Kai-ying Zhang**^2, *^**, Bin-bin Li**^1^**,** **Xia-wan Liu**^2^**, Jing-rong Li**^2^**, Yi-nuo Liu**^2^**, Yu-han Liu**^2^**, Ji Li**^3,^ **^†^, Xiang-shan Yuan**^2,^ **^†^, Ming Zhong**^1,4,5,^ **^†^**

^1^Department of Critical Care Medicine, Zhongshan Hospital, Fudan University, Shanghai, China

^2^Department of Anatomy and Histoembryology, School of Basic Medical Sciences, Fudan University, Shanghai, China

^3^Department of Pancreatic Surgery, Huashan Hospital, Fudan University, Shanghai, China

^4^Shanghai Key Laboratory of Lung Inflammation and Injury, Shanghai, China

^5^Shanghai Institute of Infectious Disease and Biosecurity, School of Public Health, Fudan University, Shanghai, China

*Yuan-jun Yang and Kai-ying Zhang contributed equally to this work

**^†^Correspondence**:

Ji Li (liji@huashan.org.cn)

Xiang-shan Yuan (yuanxiangshan1999@163.com)

Ming Zhong (zhong.ming@zs-hospital.sh.cn)


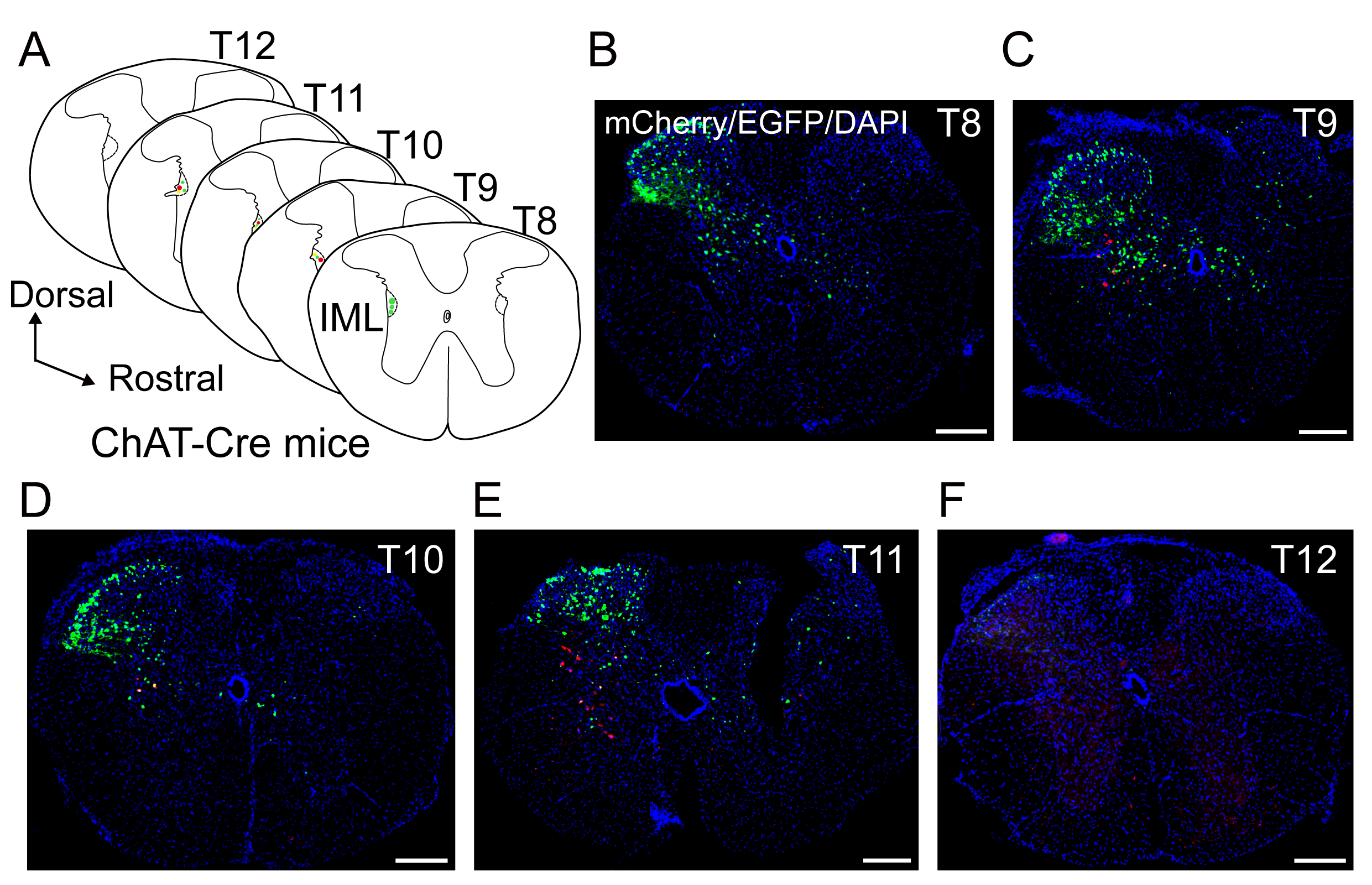
 **Supplementary Figure S1** The accuracy of the viral injection locations within the T9-T11 IML. (A) Schematic of the starter cells in the T9-T11 segments. (B-F) Representative images from T8 to T12 segments in ChAT-Cre mice.


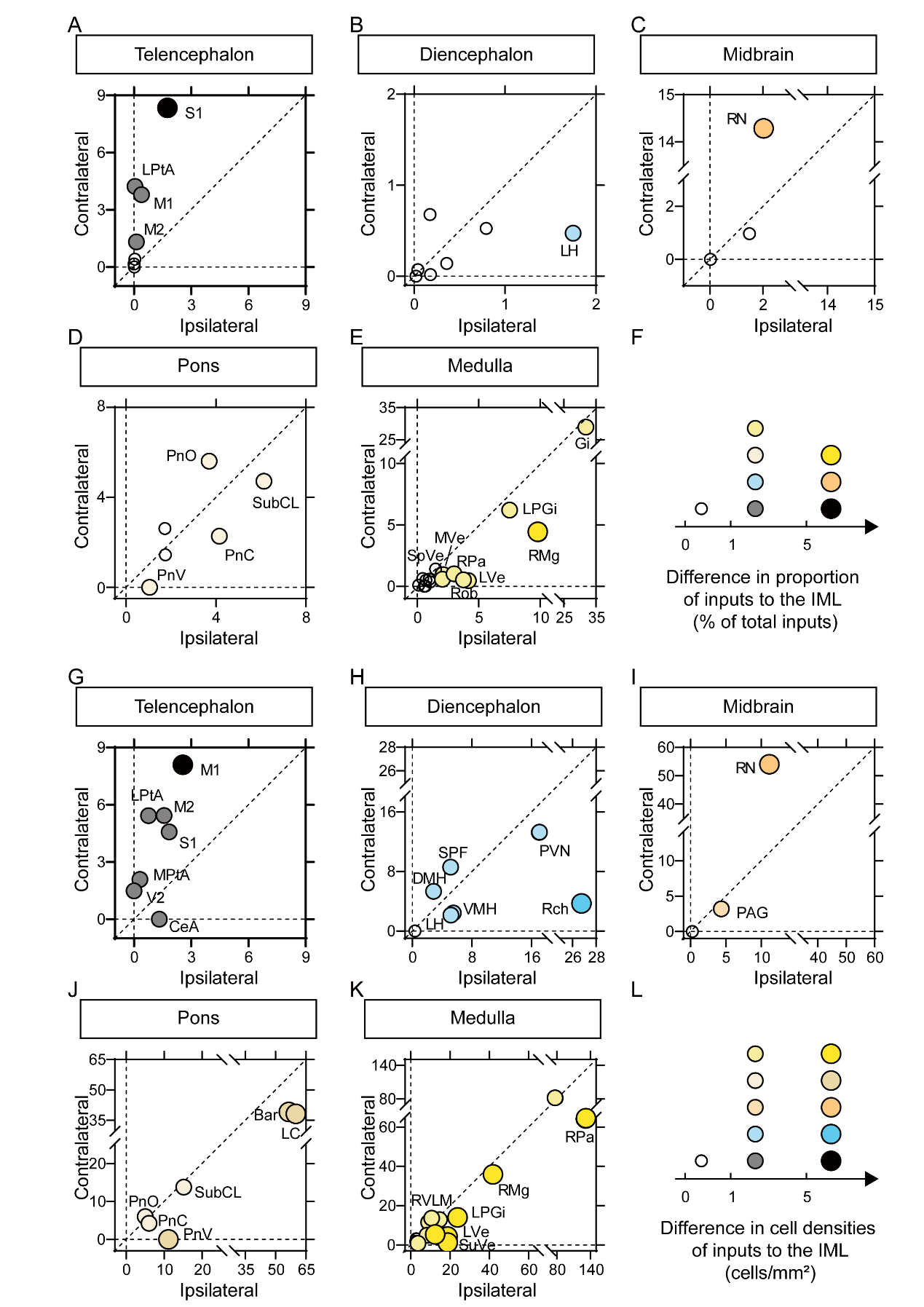
 **Supplementary Figure S2** Descriptive comparison of hemispheric distribution in supraspinal inputs to IML cholinergic neurons. (A-E) Comparative scatter plots illustrating the proportion of total inputs originating from the ipsilateral versus contralateral sides for individual nuclei. Data are segmented by major brain divisions: Telencephalon (A), Diencephalon (B), Midbrain (C), Pons (D), and Medulla (E). Each data point represents a specific brain region. The diagonal line serves as a reference for bilateral symmetry. (F) Legend for panels A-E. The visual style of the data points categorizes the magnitude of the inter-hemispheric difference in projection proportion: empty circles represent a difference of 0-1%, light-colored circles represent 1-5%, and dark-colored circles represent >5%. (G-K) Comparative scatter plots illustrating the cell density (cells/mm²) of afferent inputs from the ipsilateral versus contralateral sides. Panels correspond to the Telencephalon (G), Diencephalon (H), Midbrain (I), Pons (J), and Medulla (K). (L) Legend for panels G-K, and the circle styles indicate the magnitude of the inter-hemispheric difference in cell density. Points deviating from the diagonal line highlight nuclei with observable hemispheric variation in either proportion or density.
